# Chromosome‐level genome assembly for the horned‐gall aphid provides insights into interactions between gall‐making insect and its host plant

**DOI:** 10.1002/ece3.8815

**Published:** 2022-04-21

**Authors:** Hong‐Yuan Wei, Yu‐Xuan Ye, Hai‐Jian Huang, Ming‐Shun Chen, Zi‐Xiang Yang, Xiao‐Ming Chen, Chuan‐Xi Zhang

**Affiliations:** ^1^ Key Laboratory of Breeding and Utilization of Resource Insects National Forestry and Grassland Administration Institute of Highland Forest Science Chinese Academy of Forestry Kunming China; ^2^ 47862 Institute of Insect Sciences Zhejiang University Hangzhou China; ^3^ State Key Laboratory for Managing Biotic and Chemical Threats to the Quality and Safety of Agro‐products Key Laboratory of Biotechnology in Plant Protection of MOA of China and Zhejiang Province Institute of Plant Virology Ningbo University Ningbo China; ^4^ 5308 Department of Entomology Kansas State University Manhattan Kansas USA

**Keywords:** chromosome‐level genome assembly, gall formation, PacBio sequencing, *Schlechtendalia chinensis*

## Abstract

The aphid *Schlechtendalia chinensis* is an economically important insect that can induce horned galls, which are valuable for the medicinal and chemical industries. Up to now, more than twenty aphid genomes have been reported. Most of the sequenced genomes are derived from free‐living aphids. Here, we generated a high‐quality genome assembly from a galling aphid. The final genome assembly is 271.52 Mb, representing one of the smallest sequenced genomes of aphids. The genome assembly is based on contig and scaffold N50 values of the genome sequence are 3.77 Mb and 20.41 Mb, respectively. Nine‐seven percent of the assembled sequences was anchored onto 13 chromosomes. Based on BUSCO analysis, the assembly involved 96.9% of conserved arthropod and 98.5% of the conserved Hemiptera single‐copy orthologous genes. A total of 14,089 protein‐coding genes were predicted. Phylogenetic analysis revealed that *S*. *chinensis* diverged from the common ancestor of *Eriosoma lanigerum* approximately 57 million years ago (MYA). In addition, 35 genes encoding salivary gland proteins showed differentially when *S*. *chinensis* forms a gall, suggesting they have potential roles in gall formation and plant defense suppression. Taken together, this high‐quality *S*. *chinensis* genome assembly and annotation provide a solid genetic foundation for future research to reveal the mechanism of gall formation and to explore the interaction between aphids and their host plants.

## Graphical abstract

The genome of a galling aphid *Schlechtendalia chinensis* is 271.52 Mb. Nine‐seven percent of the assembled sequences were anchored onto 13 chromosomes. A total of 14,089 protein‐coding genes were predicted and 35 genes encoding salivary gland proteins have potential roles in gall formation and plant defense suppression.

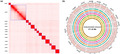

## 1 INTRODUCTION

Numerous aphid species are economically important plant pests that feed on plant sap. Many plant‐feeding aphids can also transmit plant viruses. Around 100 out of approximately 5000 known aphid species are significant agricultural pests due to their feeding damages and/or disease transmission (Blackman & Eastop, 2020). Currently, studies on aphid genomes have mainly focused on the subfamily Aphidinae (International Aphid Genomics Consortium, 2010; Li, Zhao, et al., 2019; Li et al., 2019; Mathers, 2020; Mathers et al., 2017, 2020; Mathers, Wouters, et al., 2020; Nicholson et al., 2015; Thorpe et al., 2018; Wenger et al., 2016). Genome sequencing on species from other subfamilies that are distantly related to Aphidinae is relatively limited (Biello et al., 2021; Julca et al., 2020). Unlike most free‐living aphids, galling aphids can induce gall formation on their primary host plants and then live in galls. Galling aphids may are ideal models to study unique ecological and behavioral phenomena underlying insect–plant interactions and their coevolution (Moran, 1989; Wool, 2004). So far, only two galling aphids, *Eriosoma lanigerum* and *Hormaphis cornu*, have been sequenced and assembled. One species,  *E*. *lanigerum,* often causes bark deformation and cancer‐like swelling on the roots, trunk or brunches of apple, and sometimes induces the formation of leaf‐rosette galls on American elm (*Ulmus americana*) (Blackman & Eastop, 2020). Another species, *H*. *cornu*, induces a gall on the underside of leaves of witch hazel, *Hamamelis virginiana* (Kurosu et al., 1992). However, the galls induced by *E*. *lanigerum* and *H*. *cornu* are quite different from the completely closed galls induced by *Schlechtendalia chinensis*, which has peculiar strategies to adapt to a closed environment that has extremely high levels of CO_2_ honeydew, and other aphid metabolites (Chen, Chen, et al., 2020; Chen, Yang, et al., 2020).

The horned gall aphid, *S*. *chinensis* (Hemiptera: Aphididae: Eriosomatinae: Fordini), is one of the most economically valuable insects. Gallnuts induced by the aphids are valuable for medicinal purposes and in chemical industries. The components in gallnuts, such as tannins, are important gradients for producing inks, wine, food, cosmetic antioxidants, and animal feed. High levels of tannins (50%–70%) have been found in horned galls (Zhang et al., 2008). The annual yield of gallnuts in China is 8000–10,000 tons, accounting for over 90% of the total yield worldwide (Zhang et al., 2008).

**FIGURE 1:**
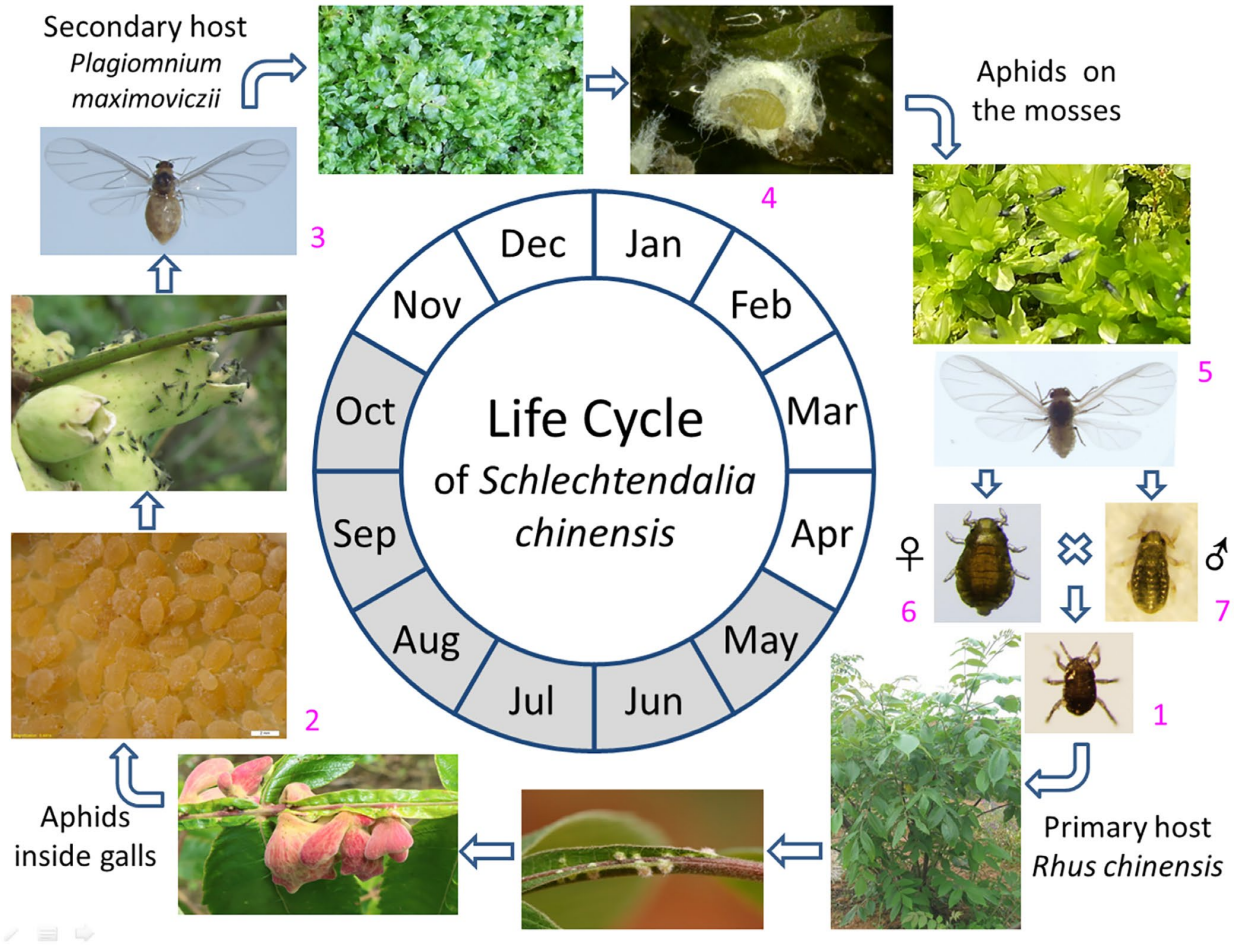
Life cycle diagram of *Schlechtendalia chinensis*. A typical life cycle of the horned gall aphid in Hubei, China. A fundatrix (1) finds a suitable tender leaf on the primary host *Rhus chinensis*, to feed and initialize gall formation, and feeds inside the induced gall by the end of April or the beginning of May. Afterward, the fundatrix and the wingless daughters (called fundatrigeniae) (2) reproduce for generations viviparously and parthenogenetically within the gall from May to October. The gall size increases gradually along with the growth of the aphid population in it. At the end of October, winged autumn migrants (3) emerge from the gall and fly away after the gall opened. The migrants find the moss *Plagiomnium maximoviczii* nearby where they produce nymphal offspring. (4) The nymphs feed on the moss and secrete waxes to wrap themselves up for overwintering. Winged spring migrants (5) emerge by the end of March, then fly back to the primary host and reproduce sexual females (6) and males (7) in the trunk crevices. After mating, the female reproduces a fundatrix to begin the next life cycle. *Graphs not in scale. Stippled sector indicating the in‐gall stages

*Schlechtendalia chinensis* has a complex life cycle involving both sexual and asexual reproduction stages with a host alternation between the Chinese sumac (*Rhus chinensis*, Anacardiaceae) and mosses of the genus (*Plagiomnium* spp., Mniaceae). In this holocyclic life cycle, a fundatrix produced by a mated female crawls along the trunk and feeds on a new leaf, where it induces the formation of a horned gall. The fundatrix can produce wingless fundatrigeniae in galls via parthenogenesis. In autumn, wingless fundatrigeniae will produce winged fundatrigeniae named autumn migrants. When galls become mature and burst open, the late autumn migrants will fly to nearby mosses and produce nymphs for overwintering. In the following spring, nymphs on mosses will develop into spring winged migrants, which then fly back to the primary host, *R*. *chinensis* and produce both female and male offspring called sexuales. After mating, each female reproduces only one fundatrix, starting the cycle again (Figure 1) (Blackman & Eastop, 2020; Zhang et al., 1999). This represents an unusual life cycle with comprising various morphologically distinct aphid forms at different stages, and its evolution was likely driven by the adaptation to different environmental conditions. Unlike most free‐living aphids from the Aphidinae taxon, galling aphids exhibit diverse biological characteristics. For example, most galling aphid species do not seriously affect the health of their host plants. In some cases, the galls are thought to be beneficial to host plants (Chen, Chen, et al., 2020; Chen, Yang, et al., 2020).

For *Schlechtendalia chinensis*, the complexities in its developmental process and the structure of its induced galls imply that it may possess unique gene sets that regulate its development and manipulate its host plants (Hirano et al., 2020; Takeda et al., 2019). The molecular mechanisms underlying its complex life cycle remain largely unknown. Galls are produced through the insect‐driven dramatic reprogramming of plant cell biology. Previous studies have shown that gall induction is highly species‐specific, and that different galling insects deliver unique sets of effectors into plant tissues, resulting in gall formation (Aljbory et al., 2018; Zhao et al., 2015). The underlying mechanisms of the parasitic ability of galling aphids on host plants via apparently harmless galls remain unknown so far. To understand the genetic basis of the complex lifestyle, a high‐quality chromosome‐level genome assembly of *S*. *chinensis* accomplished, representing the first genome sequence of aphids that induces the formation of completely closed galls. Phylogenetic relationship between *S*. *chinensis* and closely related species was analyzed to better understand the unique biological characteristics of *S*. *chinensis*.

## 2 MATERIALS AND METHODS

### 2.1 Sample collection

*Schlechtendalia chinensis* samples were collected from fresh mature galls on *R*. *chinensis*, in Wufeng county (30°10′ N, 110°52′ E, 960 m above sea level), Hubei Province China, on October, 2019. A colony was established through artificial cultivation for further genetic studies. Briefly, autumn migrants of *S*. *chinensis* from mature galls, transferred to a nursery of the moss *Plagiomnium maximoviczii*, and maintained in a greenhouse. In the following year, nymphs and spring migrants (sexuparae) were harvested from mosses and cultivated in laboratory. Male and female produced by spring migrants were collected in laboratory. After fundatrix emergence, aphids were transferred to host trees for gall induction. Aphid samples were collected separately at different stages, including fundatrix, fundatrigeniae, autumn migrants, overwinter nymphs, spring migrants, male and female sexuales. Fundatrigeniae (females) contained in a gall were transferred to a petri dish after dissecting the gall. Impurities like waxes were removed manually. All aphids within a gall were presumed to be the clonal offspring of a single fundatrix, since all the *S*. *chinensis* galls contained only one single fundatrix that produced offspring in the gall via parthenogenesis. All aphid samples were immediately frozen in liquid nitrogen for two hours and subsequently stored at −80°C until further analysis.

### 2.2 Genomic and transcriptomic sequencing

Genomic DNA (gDNA) was isolated from 200 individual female and male using the DNeasy Blood & Tissue Extraction Kit (Qiagen Inc., Valencia, CA, USA), following the manufacturer's instructions. After quality and quantity measurements, the gDNA was used to construct a 150‐bp paired‐end sequencing library for Illumina platform. A 20 kb long‐read sequencing library was constructed by gDNA isolated from 200 fundatrigeniae for PacBio Sequel II platform. For Hi‐C analysis, 200 fundatrigeniae were soaked in 1% formaldehyde for 10 min at room temperature and in a 2.5 M‐glycine solution to terminate the isolation and cross linking of aphid cells. The Hi‐C assays and the sequencing procedures were performed via a commercial contract with Annoroad Gene Technology Co., Ltd. (Beijing, China) (Rao et al., 2014).

Transcriptomes were generated from RNA samples extracted from different stages including fundatrix, fundatrigeniae, autumn migrants, nymphs, spring migrants (sexuparae), male and female sexuales, separately. RNA quantity, purity and integrity were determined on a NanoPhotometer and an Agilent 2100 Bioanalyzer. cDNA libraries were subsequently constructed following the chain specific method. The libraries were initially quantified by the qubit 2.0 fluorometer and diluted to 1.5 ng/µl. Later, different libraries were pooled according to the requirements of effective concentration and target data volume for Illumina sequencing. Low‐quality bases in the RNA‐Seq raw reads were filtered using Trimmomatic (version 0.36) (Bolger et al., 2014). Clean reads were mapped to the genome assembly using Hisat2 (version 2.1.0.5) (Kim et al., 2015), so as to obtain the putative transcripts. Transcript levels were analyzed using cufflinks (version 2.2.1) (Ghosh, & Chan, 2016).

### 2.3 Genome assembly

The Illumina paired end reads were used for k‐mer analysis to estimate the genome size and heterozygosity with a k‐mer length of 17 bases. Specifically, the k‐mer number and distribution were calculated based on Jellyfish (version 1.1.10, parameters set to ‐C, ‐m 17, ‐s 10G, ‐t 80), whereas the genomic information was counted and visualized using GenomeScope (version 2.0, parameters set to 12, 150) (Marçais & Kingsford, 2011; Ranallo‐Benavidez et al., 2020). Pacbio sequencing data were used to assemble the draft genome using Wtdbg2 (version 2.5, parameters set to ‐t 8, ‐p 21, ‐S 4, ‐s 0.05, ‐g 274m, ‐L 5000) (Ruan & Li, 2020). Potential sequences from bacteria, fungi and other microorganisms were removed by aligning the genome sequences to the Nt database. Both long and short reads were utilized to correct base errors in the draft genome using NextPolish (Hu et al., 2019). HaploMerger2 (with default parameters) and purge_haplotigs (parameters set to ‐m 4G; ‐t 60; ‐l value1, ‐m value2, ‐h value3; ‐t 60, ‐a 70) were adopted to remove the heterozygous regions in the genome (Huang et al., 2017; Roach et al., 2018).

To construct the chromosome‐level genome assembly, Hi‐C sequences were aligned to the haploid genome assembly using Juicer (version 1.5, with default parameters). An initial chromosome‐level assembly was generated via the 3D de novo assembly (3D‐DNA) (version 180114) analysis with the parameter “‐r 3” (Dudchenko et al., 2017). The final chromosome‐level assembly was reviewed using Juicebox Assembly Tools (JBAT, version 1.11.0, with default parameters) (Dudchenko et al., 2018). The completeness of genome assembly was assessed using BUSCO (v5.1.3) (Waterhouse et al., 2018) to scan the universal single‐copy orthologous genes selected from Eukaryota, Arthropoda, Insecta, and Hemiptera datasets (odb_10). The final assembly was validated based on the Illumina reads and RNA sequencing (RNA‐seq) reads via bowtie2 (Table S1).

### 2.4 Localization of the sex chromosomes and autosomes

The mapped reads per million (MRPM) of each chromosome for female and male Illumina reads were calculated to locate the sex chromosomes and autosomes (Ye et al., 2021). The normalized read counts of the X chromosome are approximately twice higher in females than those in males, because males have only one copy of the X chromosome, while female have two copies. Both males and females have two copies in the autosomes, and the ratio of males and females is expected to approach 1 (Pal & Vicoso, 2015). Male and female DNA reads were mapped separately to the genomic scaffolds using Bowtie2 with default parameters (Langmead & Salzberg, 2012). The resulting alignments were later filtered to remove the low‐quality mapped reads via SAMtools view (‐b ‐q 30). The read counts of each chromosome were calculated using SAMtools idxstats (Li et al., 2009). The sex chromosomes were then verified by comparison with other species. Syntenic blocks of genes were identified between the chromosome‐level genome assemblies of *S*. *chinensis*, *Acyrthosiphon pisum*, *Rhopalosiphum maidis*, *E*. *lanigerum* by adopting MCSCANX and visualization via Dual Systeny Plotter for MCSCANX of the synteny visualization of TBtools (version 1.09, Chen, Chen, et al., 2020; Chen, Yang, et al., 2020) (Table S1).

### 2.5 Gene annotation

To predict the repetitive regions, RepeatMasker (version 4.1.1) (Tarailo‐Graovac & Chen, 2009) was employed to screen the *S*. *chinensis* genome against the Repbase library (Bao et al., 2015), and the parameter was set to RepeatMasker ‐pa 4 ‐e ncbi‐species Hemiptera ch ‐dir. Further, an aphid‐specific database was generated using RepeatModeler (version 2.0.1, with default parameters) so as to predict the transposons and repetitive regions (Flynn et al., 2020). Statistical results of RepeatMasker and Repeatmodeler analyses were combined.

Gene structures were predicted using GETA pipeline (version 2.4.2, https://github.com/chenlianfu/geta) to merge the results of the RNA‐seq assisted, homology‐based and ab initio methods. Briefly, In the RNA‐seq‐assisted method, RNA‐seq data generated from Illumina were aligned to the assembled *S*. *chinensis* genome using Hisat2 (version 2.1.0.5) (Kim et al., 2015). In the homology‐based method, genes were predicted based on homology to map protein sequences using GeneWise (version 2.4.1) (Birney et al., 2004). Augustus (version 2.5.5) (Stanke et al., 2006) was used to generate ab initio gene prediction (Alioto et al., 2018; Stanke et al., 2006). Gene prediction results were then pooled and screened against the PFAM database.

To assign functions to the newly annotated genes in the *S*. *chinensis* genome, these genes were aligned to sequences in databases including NCBI Non‐Redundant Protein Sequence (Nr), Non‐Redundant Nucleotide Sequence Database (Nt), SwissProt, Cluster of Orthologous Groups for eukaryotic complete genomes (KOG), Integrated Resource of Protein Domains and Functional Sites (InterPro), Gene Ontology (GO), Kyoto Encyclopedia of Genes and Genomes, Orthology database (KEGG), and evolutionary genealogy of genes: Nonsupervised Orthologous Groups (eggNOG). A localBlast2GO database was also built for GO annotation, which was later processed via Blast2GO (version 2.5). The KAAS of KEGG databases were utilized to annotate the *S*. *chinensis* genome sequence, and then BBH pattern was chosen.

### 2.6 Non‐coding RNA identification

Transfer RNAs (tRNAs) were identified using the tRNAscan‐SE program (version 1.3.1, with default parameters for eukaryotes) (Chan & Lowe, 2019). RNAmmer (version 1.2, parameters set to “‐s euk ‐m tsu, ssu, lsu”) was used to identify 5S/8S, 16S/18S and 23S/28S ribosomal RNAs (rRNAs) (Karin et al., 2007). rRNAs, microRNAs (miRNAs) and small nuclear RNAs (snRNAs) were identified based on the Rfam database (version 12.2) using BLASTN (E‐value ≤1 × 10^−5^) (Kalvari et al., 2018).

### 2.7 Phylogenetic analysis

Phylogenetic trees for *S*. *chinensis* and eight other aphid species including *Daktulosphaira vitifoliae*, *Sipha flava*, *Aphis glycines*, *R*. *maidis*, *A*. *pisum*, *Myzus persicae*, *Diuraphis noxia*, *E*. *lanigerum* were reconstructed (International Aphid Genomics Consortium, 2010; Li, Zhao, et al., 2019; Li, Park, et al., 2019; Mathers, 2020; Mathers et al., 2017; Mathers, Mugford, et al., 2020; Mathers, Wouters, et al., 2020; Nicholson et al., 2015; Thorpe et al., 2018; Wenger et al., 2016). The whitefly, *Bemisia tabaci* was used as the outgroup. The aphid genome sequence and gene structure annotation files were downloaded from the NCBI genome database, genes containing mRNA information were retained, and the CDS was modified. The longest isoform was selected as the representative sequence of the gene. Predicted proteins encoded by all putative genes were obtained. Orthologous groups were assigned by OrthMCL (v2.0.9) (Li et al., 2003) based on the all‐versus‐all BLASTP results (E‐value ≤1 × 10^−5^). Single copy orthologous groups were extracted from OrthoMCL results where single copy genes covered at least 50% of all species. And if the shortest sequence of the single copy ortholog group is longer than 6000 bp, the single copy ortholog group is filtered out to avoid too long sequences that may affect the accuracy of tree. Multi‐sequence alignments of single copy orthologous genes were performed using MAFFT (version 7.221, Katoh et al., 2002; Katoh & Standley, 2013) and the conserved amino‐acid sites were identified using Gblocks (version 0.91, Clore, 2014). RAxML (version 8.1.24) (Stamatakis, 2014) was employed to construct the phylogenetic tree under the GTRGAMMA model with 1000 bootstrapping replicates (Castresana, 2000). The branch length of homologous genes was analyzed with PAML (Yang, 2007), and compared with the standard tree to eliminate abnormal genes. Then, the tree was rebuilt using RAxML again (Stamatakis, 2014). By providing the root number and multiple sequence alignment results with calibration point information, the species divergence time was calculated using MCMCtree of PAML software (version 14.9). Divergence time within the evolutionary tree was obtained with 95% confidence interval (CI) (Yang, 2007). Meanwhile, divergence time and age of fossil records were derived from TimeTree (http://www.timetree.org/) and applied as the calibration points. According to the divergence times from TimeTree, the nodal dates of *Ac*. *pisum* and *Ap*. *glycines* were 28–61 million years ago (MYA), those of *D*. *vitifoliae* and *S*. *flava* were 87–162 MYA and those of *B*. *tabaci* and *D*. *vitifoliae* were 245–351 MYA (Johnson et al., 2018).

### 2.8 Gene family expansion and contraction

CAFE (version 3.1) (Hahn et al., 2007) was used to analyze gene family expansion and contraction by comparing the *S*. *chinensis* genome with those from eight other aphid species (namely *D*. *vitifoliae*, *S*. *flava*, *E*. *lanigerum*, *Ap*. *glycines*, *R*. *maidis*, *Ac*. *pisum*, *D*. *noxia* and *M*. *persicae*). Briefly, the quantitative information of gene families of 10 insects was obtained based on the OrthoMCL results. The number of gene families in each species and the trees with divergence time were used as the input information of CAFE (parameters set to “lambda ‐s, ‐t”). The best rates for gene birth and death were decided using CAFE, and all branches had the same rates of gene birth and death. Expansion and contraction of gene families were identified using CAFE (Hahn et al., 2007). GO and KEGG enrichment analyses were conducted using Omicshare CloudTools under default instructions settings (http://www.omicshare.com/).

### 2.9 Identification of genes potentially involved in gall formation and host manipulation

One hundred and forty‐one proteins were identified from the saliva of *S*. *chinensis* in a previous study (Yang et al., 2018). These identified proteins were used to identify genes potentially involved in gall formation and host manipulation. tBLASTN was used to search the corresponding genes in the *S*. *chinensis* genome with the 141 salivary proteins as queries (E‐value ≤1 × 10^−5^, identify ≥50). The expression levels of salivary protein‐encoding genes were quantified in three stages based on the RNA‐seq data. Upregulated genes in fundatrix were subject to GO and KEGG enrichment analyses using Omicshare CloudTools with default parameters (http://www.omicshare.com/).

## 3 RESULTS AND DISCUSSION

### 3.1 Genome sequencing and *de novo* assembly

The k‐mer (*K* = 17) analysis indicated that the heterozygosity of *S*. *chinensis* was 0.786% and the estimated genome size was 274,512,001 bp (Figure S3). The sequencing of the fundatrigenia genome (using the PacBio Sequel II platform) generated 130 Gb raw data with an N50 length of 21,033 bp. The raw contig‐level assembly was composed of 304,774,269 bases with 1409 contigs and the N50 length of 2,961,835 bp (Table 1). After removing the heterozygosity, the length of final contig‐level assembly was 271,416,320 bp with 378 contigs, and N50 length of 4,333,385 bp (Table 1).

**TABLE 1:** Statistics of the *Schlechtendalia chinensis* genome assembly

Stat Type	PicBio subreads	The draft genome assembly		The initial assembly	
		RAW contigs	Haploid contigs	Chromosomes	Small scaffolds
Base pairs	127,473,721,171	304,774,269	271,416,320	263,858,029	7,666,804
Number of contigs	9,286,929	1409	378	223	341
Contig N50	21,033	2,961,835	4,333,385	3,766,587	50,000
Number of scaffolds				13	341
Scaffold N50				20,405,002	50,000

**FIGURE 2:**
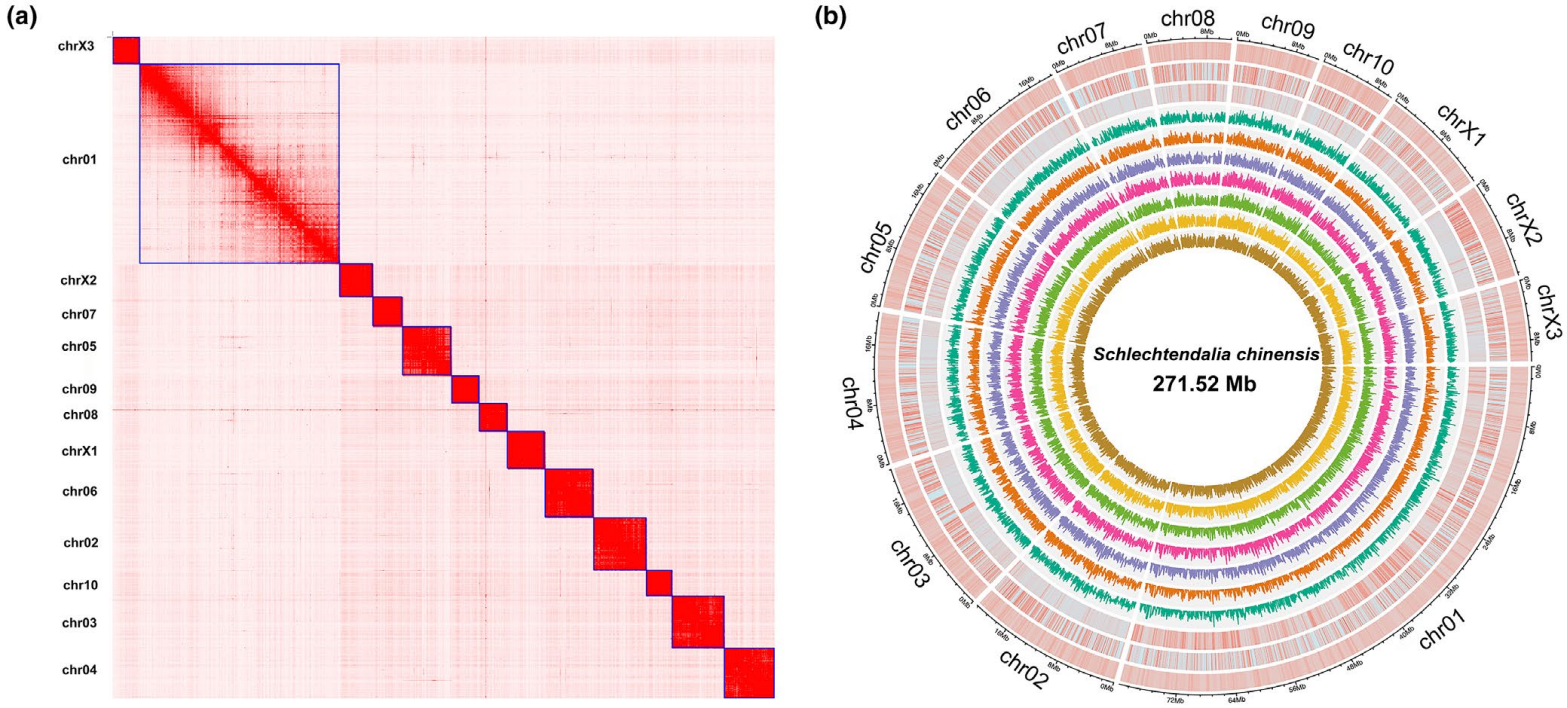
Chromosomal analysis of *Schlechtendalia chinensis*. (a) Contact maps of Hi–C interactions among chromosomes in the *S*. *chinensis* genome. The heatmap was generated by Juicebox software using in situ Hi‐C data (the resolution is 300 kb). (b) From the outside toward the inside, the first circle shows the 13 chromosomes. The second circle shows GC contents. The third circle represents repeat density across the genome. The fourth circle displays gene density across the genome. The fifth to eleventh circles show autumn migrant, fundatrix, fundatrigenia, nymph, spring migrant, male, and female

The chromosome‐level genome was generated via Hi‐C data (Table S1) with a total length of 271,524,833 bp, with a scaffold of N50 20,405,002 (Table 1). More than 97% of the total genome bases were successfully anchored to the 13 chromosomes (Figure 2). The remaining 2.8% sequences were comprised 341 small scaffolds (Table 1). Chromosome lengths ranged from 14,859,000 bp to 10,104,278 bp. As revealed by BUSCO analyses against the Eukaryota, Arthropoda, Insecta, and Hemiptera datasets, the *S*. *chinensis* genome assembly contained a higher number of conserved single‐copy Arthropoda genes than any other published aphid genome, suggesting the completeness and high quality of our genome assembly (Figure 4a). The genomic short reads were mapped to the assembled genome sequences, resulting in a 97.79% mapping rate and 60 Gb average sequence depth (Table S2). RNA‐seq isolated from seven samples including fundatrix, fundatrigeniae, autumn migrants, nymphs, spring migrants (sexuparae), and male and female sexuales, a total of 124.22 Gb raw data were generated using the Illumina platform, and more than 86% of the assembled RNA‐seq transcripts were mapped to the genome (Table S3). Altogether 260,508 transcripts (280,520,495 bp in total) were produced by Trinity (Table S4).

### 3.2 Sex chromosomes and autosomes

Male and female Illumina paired‐end reads were mapped separately to genomic scaffolds to estimate MRPM. The MRPM values of female reads for chrX1, chrX2 and chrX3 were 1,439,092, 1,333,387 and 1,051,602, whereas those for the corresponding male reads were 781,901, 726,210 and 576,946 respectively. As expected, MRPM values of female reads were roughly twice as high as those of male reads in chrX1, chrX2, and chrX3. For the other 10 chromosomes, no significant difference in total reads was observed between females and males, with the female‐to‐male ratio ranging from 0.90 to 1.00 (Table S5).

**FIGURE 3:**
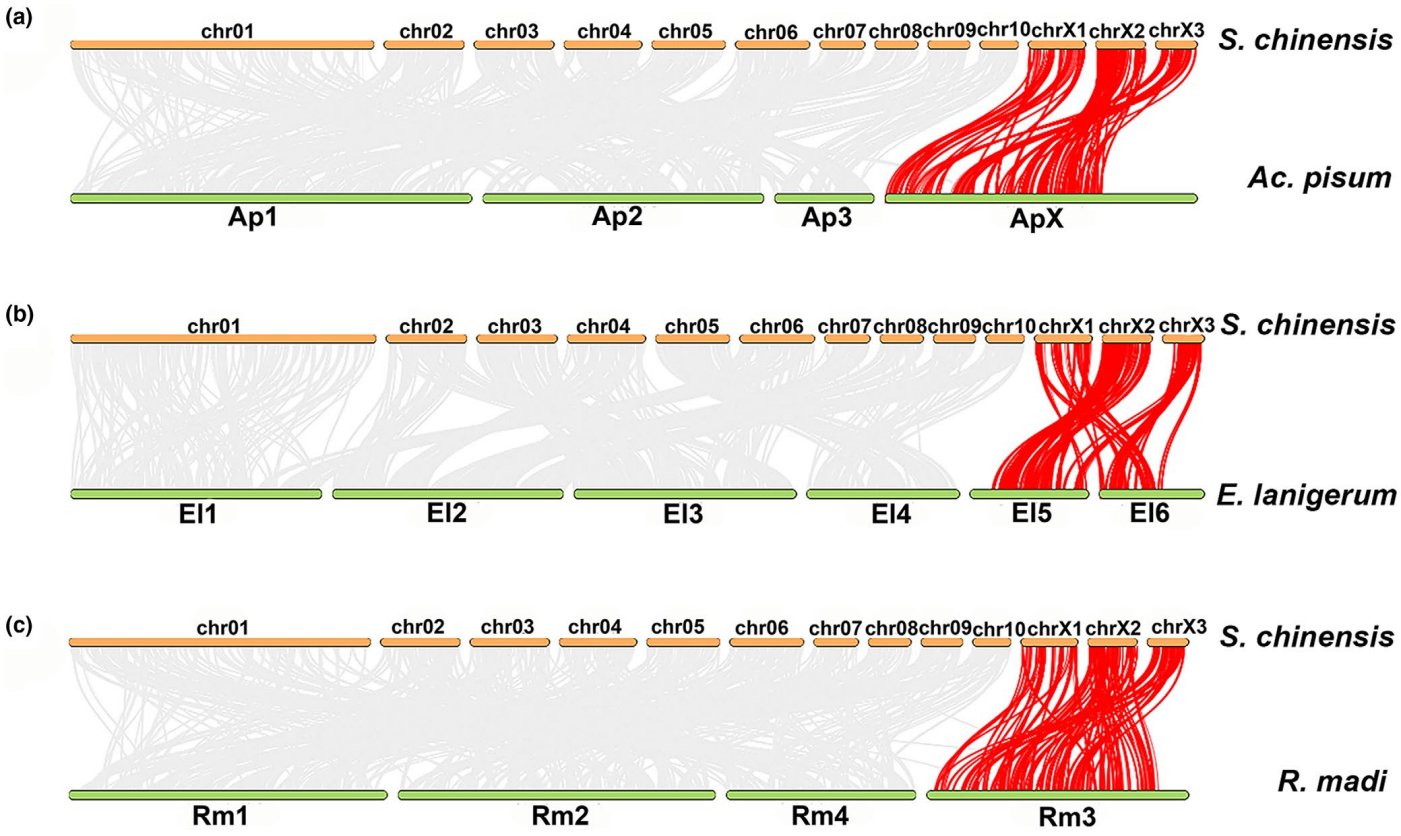
Identification of the X chromosome through syntenic blocks of chromosomal regions. Pairwise synteny relationships are shown between *Schlechtendalia chinensi*s and the chromosome‐scale genome assemblies of three aphids. (a) *Acyrthosiphon pisum*, ApX is X chromosome. (b) *Eriosoma lanigerum*, EI5 and EI6 is X chromosome. (c) *Rhopalosiphum maidis*, Rm3 is X chromosome

It has been shown that the X chromosome is conserved in aphids while chromosomal rearrangements are common for autosomes (Li et al., 2020, Mathers, Wouters, et al., 2020). The syntenic blocks were compared between the *S*. *chinensis* assembly and that of *Ac*. *pisum* from Macrosiphini (Li et al., 2020), *R*. *maidis* from Aphidini (Chen et al., 2019), and *E*. *lanigerum* from Eriosomatinae (Figure 3b). The comparisons revealed high levels of genome rearrangements between autosomes. The three *S*. *chinensis* chromosomes were mapped to the conserved X chromosome of Macrosiphini and Aphidini, and two X chromosomes of *E*. *lanigerum*. The observed multiple X chromosomes were consistent with previous reports (Biello et al., 2021), which were speculated to result from the fragmentation of the X chromosome in *S*. *chinensis* and *E*. *lanigerum* or from the ancient fusion event of the large X chromosome in Aphidinae (Macrosiphini + Aphidini). This observation strongly supports that chrX1, chrX2 and chrX3 are the sex chromosomes and the karyotype of *S*. *chinensis* is XX+X (Yuan et al., 2021).

### 3.3 Genome annotation

**FIGURE 4:**
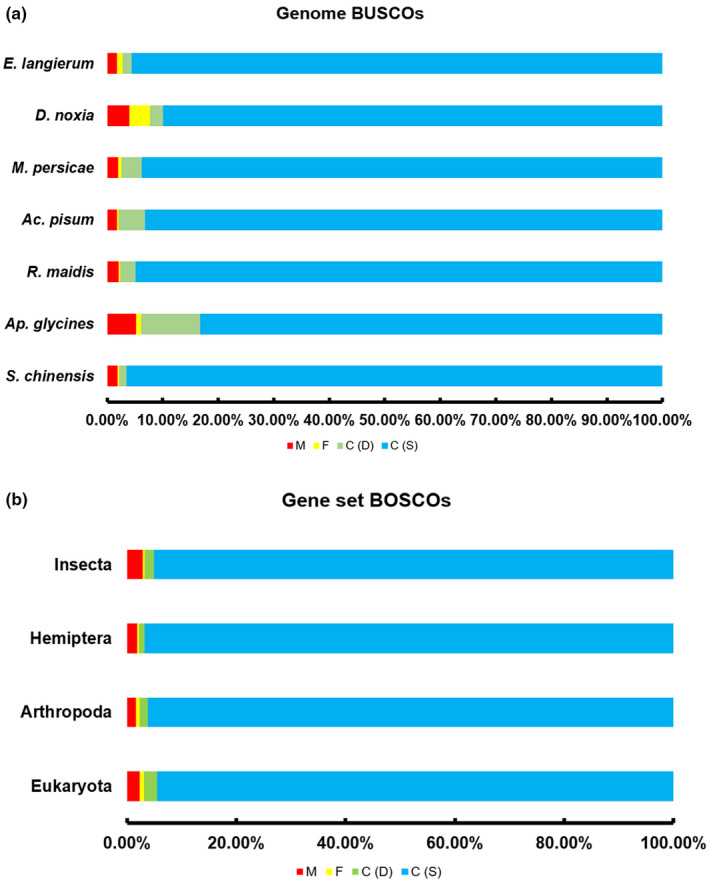
Assessments of BUSCO completeness. (a) The genome completeness values of *Schlechtendalia chinensis*, *Aphis glycines*, *Rhopalosiphum maidis*, *Acyrthosiphon pisum*, *Myzus persicae*, *Diuraphis noxia*, and *Eriosoma lanigerum* assessed by the recovery of universal single‐copy genes (BUSCOs) using the Arthropoda gene set (odb_10 and odb_9). (b) The gene set completeness of the predicted gene model of *S*. *chinensis*. The genome completeness and gene set completeness were calculated using BUSCO against Eukaryota, Arthropoda, Insecta, and Hemiptera. C, complete BUSCOs; S, complete and single‐copy BUSCOs; D, complete and duplicated BUSCOs; F, fragmented BUSCOs; M, missing BUSCOs

A total of 79,136,004 bp repetitive sequences were obtained in the *S*. *chinensis* genome, yielding a repeat percentage of 29% (Table S6). A total of 14,089 (15,987 transcripts) genes were predicted to encode proteins. There were 97.37% of the annotated genes located on the 13 chromosome‐level scaffolds (Figure 2b). The average CDS length, exon number per gene, exon length, and intron length were 1536 bp, 73,212 bp, and 910 bp, respectively, similar to those in most of the reported aphid species (Table S7, Figure S2). According to our results, 96.9%, 97.7%, 97.8%, and 96.7% of BUSCO genome/gene sets were identified in the *S*. *chinensis* genome in comparison with Eukaryota, Arthropod, Hemiptera and Insecta datasets, respectively, demonstrating the completeness of the gene set (Figure 4b). The percentage of RNA‐Seq reads assigned to a gene set was up to 80% (Table S3). Among the 14,078 predicted genes, 12,584 (89.32%) were functionally annotated, including 9272 (65.81%) genes found via GO database and 7285 (51.71%) genes via KEGG database (Table 2). Noncoding RNAs (ncRNAs) were also identified in the *S*. *chinensis* genome, including 130 tRNAs, 29 rRNAs, 29 miRNAs, and 72 snRNAs (Table S8).

**TABLE 2:** The statistics of functional annotation

Type	Number	Percent (%)
Annotation		
Nr	12,032	85.40
Nt	8680	61.61
SwissProt	7716	54.77
KOG	6018	42.71
eggNOG	10,918	77.49
vInterpro	10,582	75.11
GO	9272	65.81
KEGG	7285	51.71
Total		
Annotated	12,584	89.32
Gene	14,089	

### 3.4 Phylogenetic analysis

**FIGURE 5:**
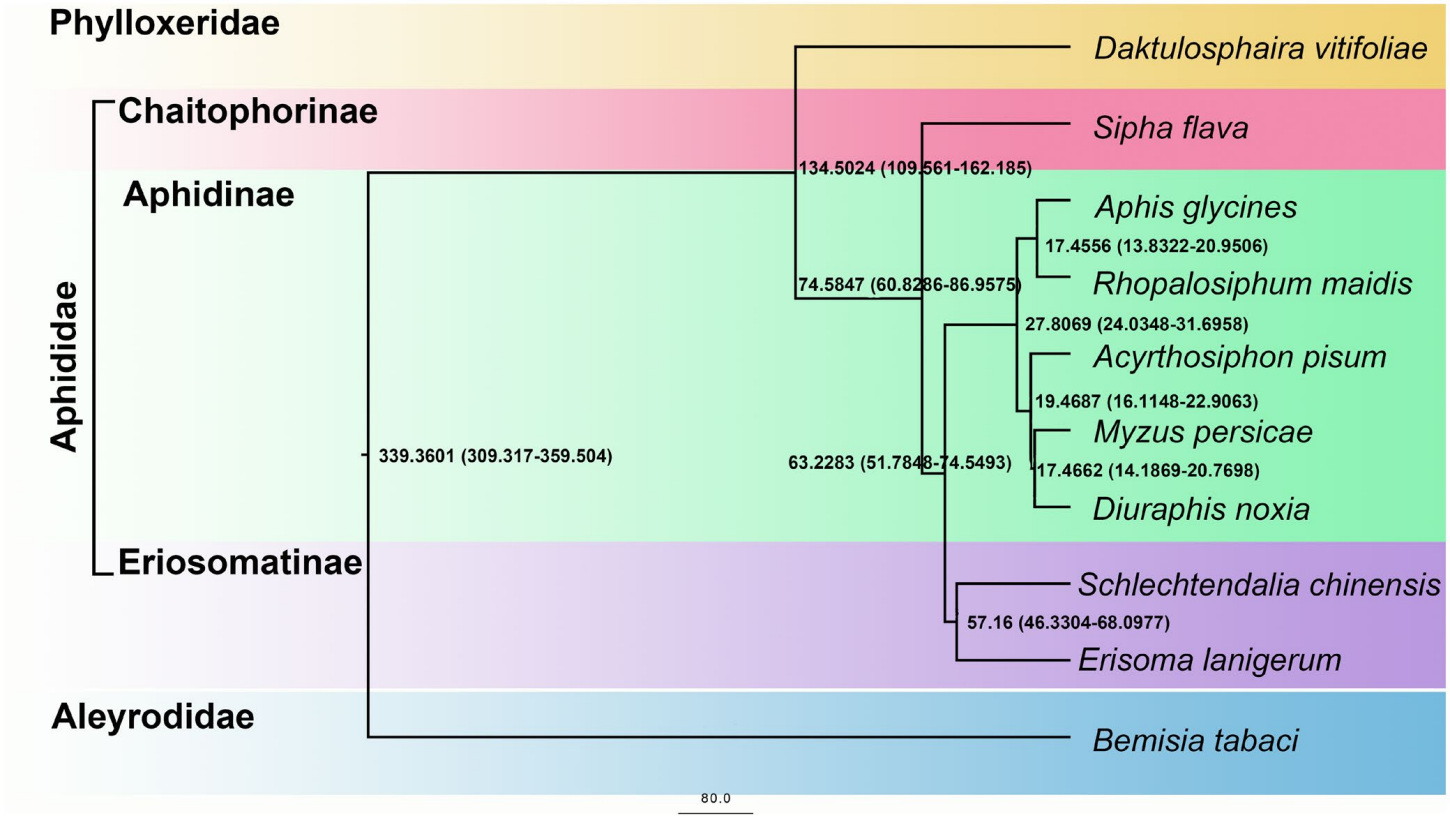
Timing of inferred divergence of *Schlechtendalia chinensis* and other nine insects

Protein sequences of *S*. *chinensis* and eight other closely related species were retrieved from public databases along, *B*. *tabaci* as an outgroup. A total of 3479 single copy orthologous groups extracted by OrthoMCL were incorporated to construct the phylogenetic tree. The results showed that *S*. *chinensis* was a sister taxon to the wooly apple aphid *E*. *lanigerum*. The two Eriosomatinae species diverged from their common ancestor at approximately 57 million years ago (MYA) (Figure 5). Eriosomatinae and Aphidinae (including *Ap*. *glycines*, *R*. *maidis*, *Ac*. *pisum*, *M*. *persicae* or *D*. *noxia*) diverged from their common ancestor at about 63 MYA, similar to the previous study (Mather et al., 2020). Compared with the subfamily Chaitophorinae (including *S*. *flava*) in the family Aphididae, the subfamily Eriosomatinae has a closer relationship with the subfamily Aphidinae. Significant expansion and contraction of gene families are usually related to the adaptive divergence of species. To elucidate the key genomic changes associated with adaptation, expansion and contraction of gene families were analyzed in all the nine aphids and *B*. *tabaci*. Eriosomatinae showed 40 expanded and 986 contracted gene families compared with those of the common ancestor of Aphidinae and Eriosomatinae (Figure S4A). KEGG and GO enrichment analyses suggested that most of the expanded genes were involved in the detoxification of natural xenobiotics from plants (Figure S4B, C). *S*. *chinensis* genome displayed 235 expanded and 1037 contracted gene families compared with of the common ancestor. KEGG pathway enrichment analysis suggested that most of the expanded gene families were involved in IL‐17 signaling pathway, arachidonic acid metabolism, NF‐kappa B signaling pathway, ovarian steroidogenesis, VEGF signaling pathway, necroptosis, regulation of lipolysis in adipocyte, TNF signaling pathway, and c‐type lectin receptor signaling pathway (Figure S4E). Similarly GO annotation analysis revealed that most of the expanded gene families were involved in prostaglandin‐endoperoxide synthase activity, arachidonate 15‐lipoxygenase activity, nucleosomes, ovarian cumulus expansion, intrinsic apoptotic signaling pathway in response to osmotic stress, regulation of fever generation, regulation of platelet‐derived growth factor production, response to lead ion, and chromatin assembly or disassembly (Figure S4D, Table S9). The expanded gene families of the *S*. *chinensis* genome were enriched not only in detoxification but also in the immune system.

### 3.5 Salivary protein‐encoding genes and other gall formation associated genes

*Schlechtendalia chinensis* can induce the formation of closed galls on host plants. Previous studies have reported that gall induction is highly species‐specific, and that galling insects deliver effectors into plant tissues, resulting in gall formation (Yang et al., 2018). The gall midge *Mayetiola destructor* can inject effector proteins into tissues via its saliva during feeding, leading to the conversion of a whole wheat seedling into a gall (Aljbory et al., 2018; Wang et al., 2018). A novel family of insect‐secreted proteins named BICYCLE has been identified in *Hormaphis cornu*, which induce gall formation on the leaves of witch hazel, *Hamamelis virginiana* (Korgaonkar et al., 2021). *BICYCLE* may regulate numerous aspects of gall development, due to their abundant expression in salivary glands specifically in gall aphids. *S*. *chinensis* feeds on host leaves where it presumably injects saliva into host leaf cells, resulting in gall formation. A total of 141 proteins have been identified from its salivary glands by LC‐MS/MS analysis (Yang et al., 2018). In comparison with salivary proteins from 10 other free‐living Hemipterans, the presence of a high proportion of proteins with binding activity is noticeable, including DNA‐, protein‐, ATP‐, and iron‐binding proteins. These proteins may be involved in gall formation. In this study, we did not identify any BICYCLE protein in the salivary glands of *S*. *chinensis*, suggesting the different mechanisms of gall induction between *S*. *chinensis* and *H*. *cornu*. As demonstrated by RNA‐Seq analysis, transcripts corresponding to 35 genes (Sc.chr03.1184–Sc.chr10.506) that encoded salivary gland proteins exhibited high expression levels in the gall forming fundatrix stage (Figure S5). These salivary proteins were potentially related to the interaction between insects and host plants. According to their predicted functions, these genes can be divided into several categories, including detoxification, signal transduction, secreted protein metabolism, energy metabolism, basic biological processes, and movement (Table S10). The largest number of genes related to detoxification may be related to defense inhibition in host plants. On the other hand, gene belonging to movement and energy metabolism categories may be associated with the contraction of salivary gland muscle and the supply of energy for salivation.

## 4 CONCLUSIONS

A high‐quality chromosome‐level genome assembly of the galling aphid *S*. *chinensis* was established in this study. Phylogenetic analysis indicated that *S*. *chinensis* diverged from *E*. *lanigerum* at approximately 57 million years ago (MYA). Transcriptome analysis showed that 35 genes that encoded salivary gland proteins were highly expressed in the gall forming fundatrix stage. Some of these salivary proteins might be involved in gall formation. Our results will benefit future research to study the molecular mechanisms underlying the unique biology associated with galling aphids, their gall induction ability, and molecular interactions between insects and plants.

## CONFLICT OF INTEREST

The authors declare that they have no competing interests.

## AUTHOR CONTRIBUTIONS

**Hong‐Yuan Wei:** Formal analysis (equal); Investigation (equal); Writing – original draft (equal). **Yu‐Xuan Ye:** Formal analysis (equal); Methodology (equal); Writing – original draft (equal). **Hai‐Jian Huang:** Methodology (supporting); Validation (supporting); Writing – review & editing (supporting). **Ming‐Shun Chen:** Methodology (supporting); Writing – review & editing (supporting). **Zi‐Xiang Yang:** Funding acquisition (lead); Project administration (equal); Writing – review & editing (equal). **Xiao‐Ming Chen:** Project administration (equal); Supervision (equal); Writing – review & editing (equal). **Chuan‐Xi Zhang:** Project administration (lead); Supervision (equal); Writing – review & editing (lead).

## Supporting information

Appendix S1

## Data Availability

All data mentioned in this paper have been deposited in the National Center for Biotechnology Information with the BioProject accession number PRJNA700780(genomic sequencing) and PRJNA702264(transcriptome sequencing). The final DNA sequence assembly has been deposited at DDBJ/ENA/GenBank under the accession JAFHKX000000000. The genome assembly and annotation, orthogroup clustering results and salivary gland genes are available for download from Zenodo (http://10.5281/zenodo.5511862).
