# Supplementary material for: Chromosome‐level genome assembly for the horned‐gall aphid provides insights into interactions between gall‐making insect and its host plant
Source: Ecol Evol. 2022 Apr 21;12(4):e8815. doi: 10.1002/ece3.8815 (PMC9021935; doi:10.1002/ece3.8815)
Supplement: Supplementary file 1 — Appendix S1 [file ECE3-12-e8815-s001.docx]

**Table of Contents:**

| **Figure S1** | Page 2 |
| --- | --- |
| **Figure S2** | Page 3 |
| **Figure S3** | Page 4 |
| **Figure S4** | Page 5-6 |
| **Figure S5** | Page 7-8 |
| **Table S1** | Page 9 |
| **Table S2** | Page 9 |
| **Table S3** | Page 9 |
| **Table S4** | Page 9-10 |
| **Table S5** | Page 10 |
| **Table S6** | Page 10-11 |
| **Table S7** | Page 11 |
| **Table S8** | Page 11 |
| **Table S9** | Page 11-13 |
| **Table S10** | Page 13-14 |


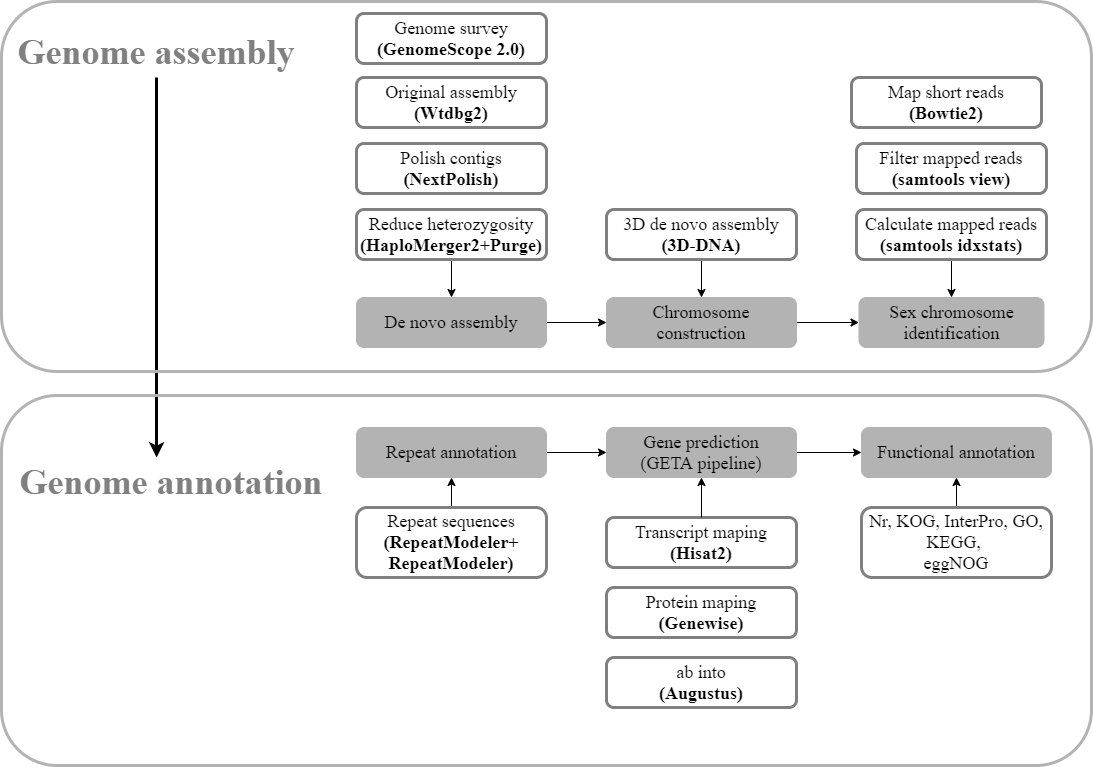


**Figure S1**. The overview of the method of genome assembly and annotation


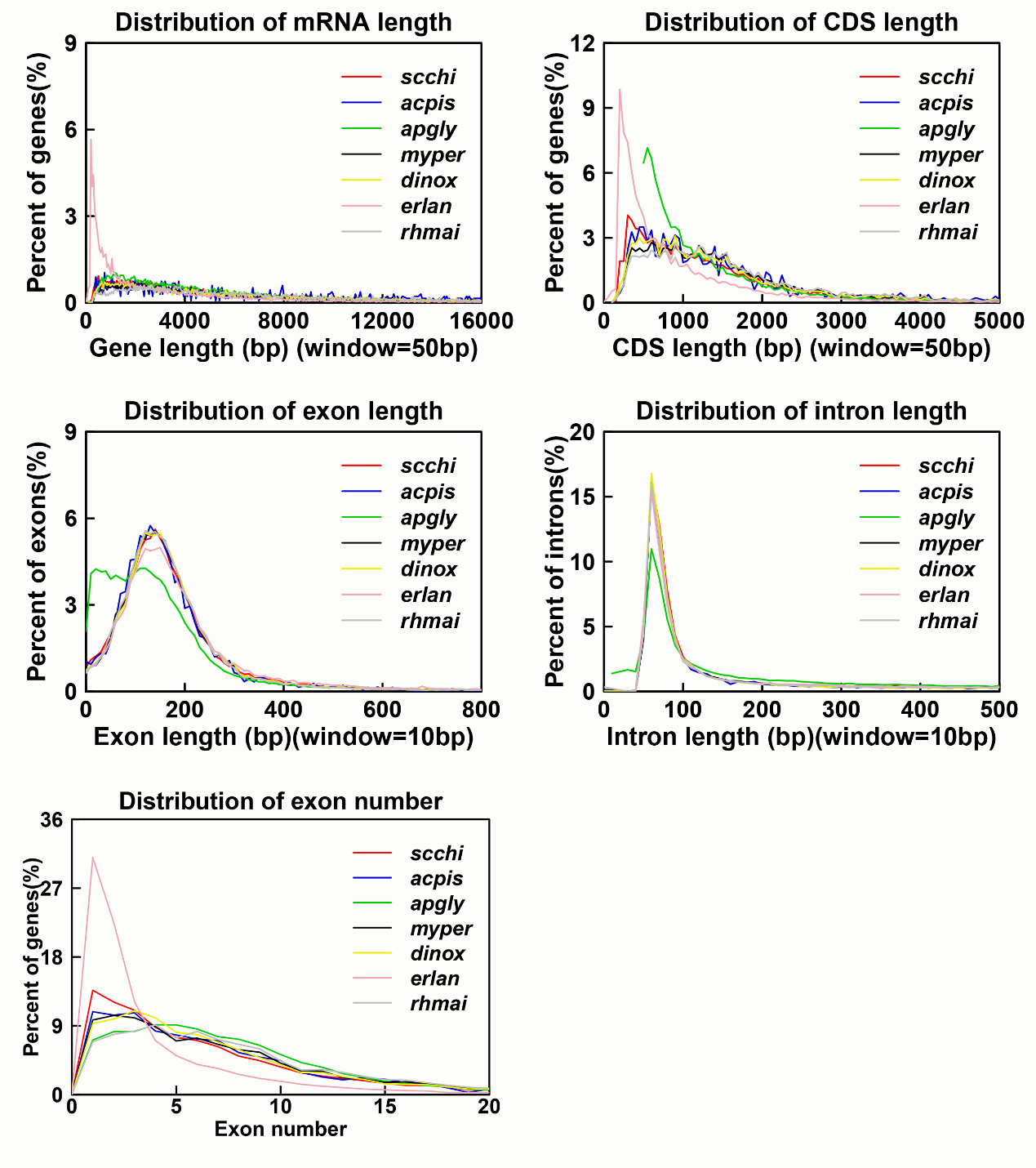


**Figure S2**. Characteristics of the annotated protein-coding genes in the *Schlechtendalia chinensis* genome

scchi: *Schlechtendalia chinensis*, acpis: *Ac. pisum*, apgly: *Ap. glycines*, myper: *M. persicae*, dinox: *D. noxia*, erlan: *E. lanigerum*, rhmai: *R. maidis*.


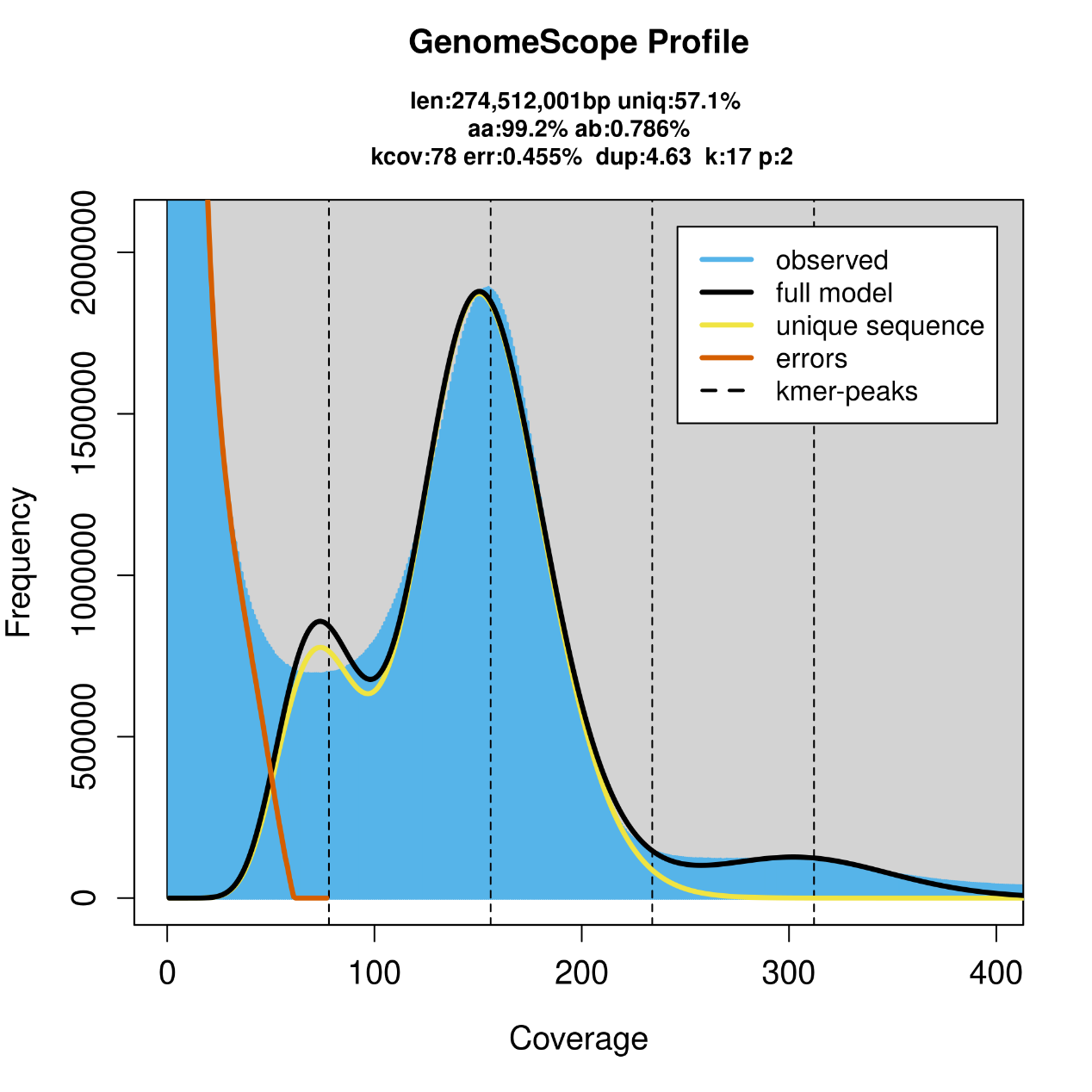


**Figure S3.** The K-mer distribution of Illumina paired-end reads using GenomeScope based on a k value of 17

Homozygous (aa): 99.18% - 99.24%; heterozygous (ab): 0.76% - 0.81%; genome haploid length (len): 273,458,379 bp - 274,512,001 bp


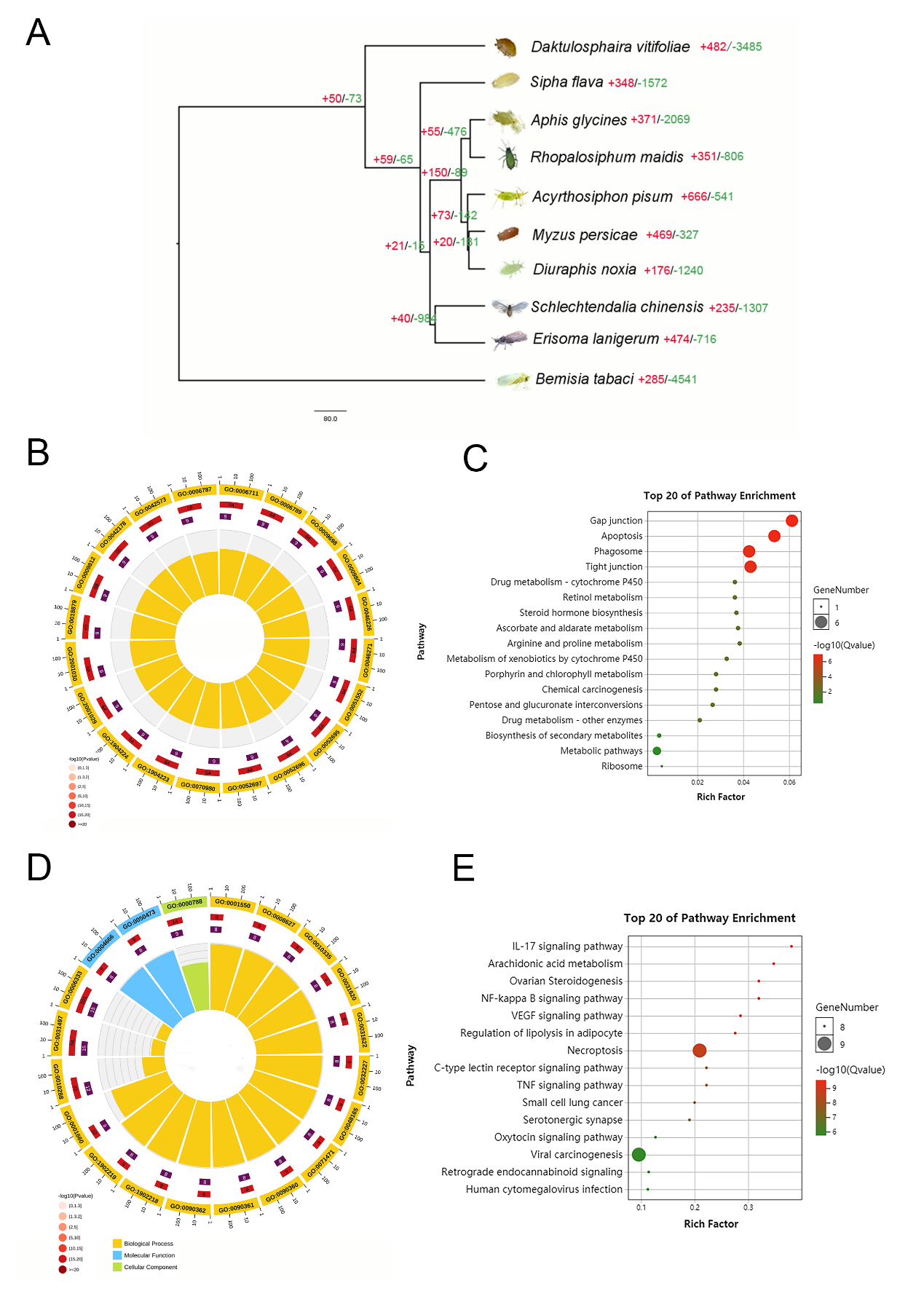


**Figure S4**. Gene family evolutions in *Schlechtendalia chinensis* and other nine insects

A. Gene family expansion (indicate in red) and contraction (indicate in green) in *S. chinensis* and other nine insects. B. Gene ontology (GO) enrichment analysis of expanded gene families of Eriosomatinae. C. KEGG pathway enrichment analysis was performed for the expansion gene family of Eriosomatinae. D. Gene ontology (GO) enrichment analysis of expanded gene families of *S. chinensis*. E. KEGG pathway enrichment analysis was performed for the expansion gene family of *S. chinensis*.


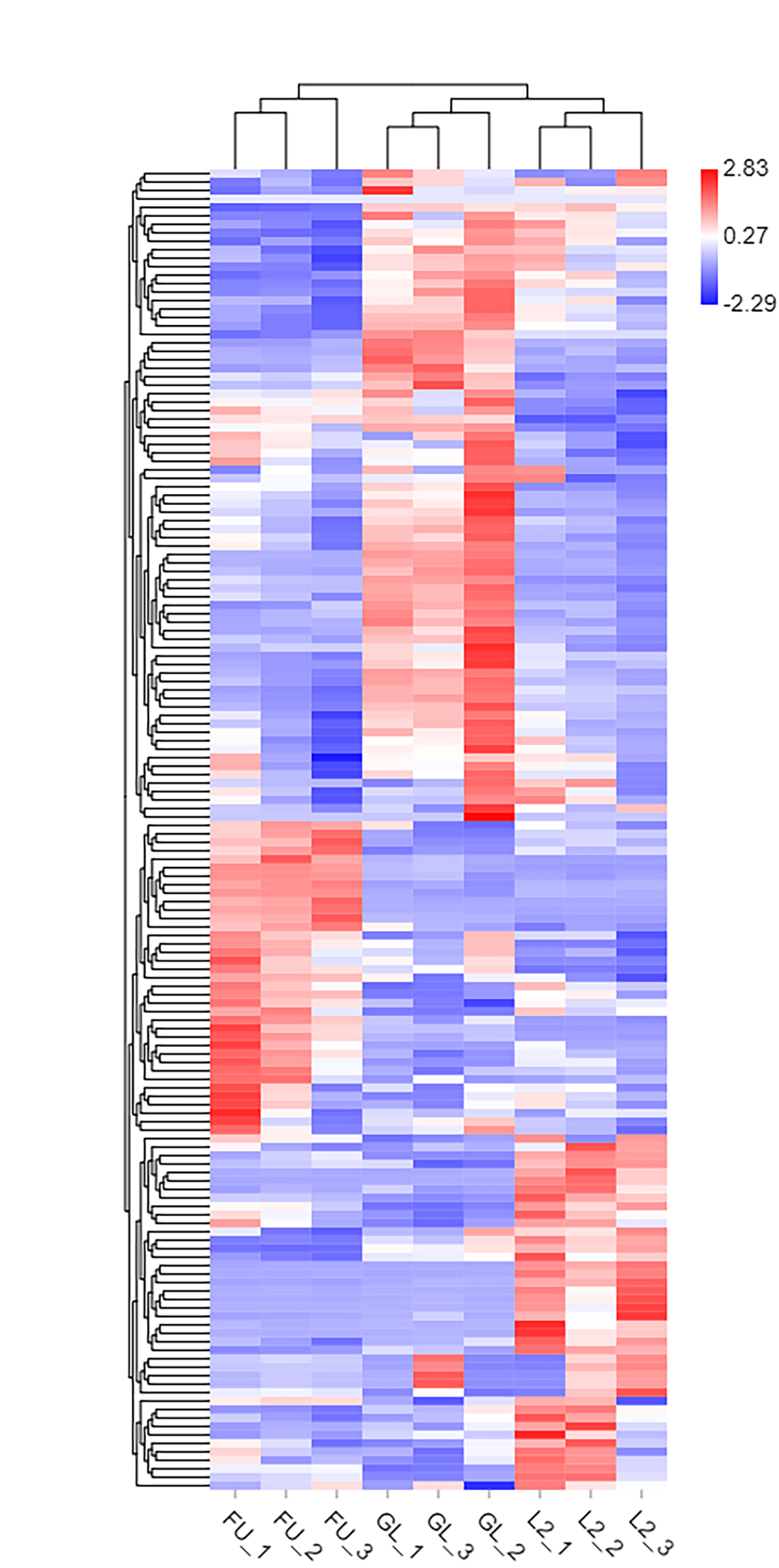


**Figure S5**. Analysis of salivary proteins of *Schlechtendalia chinensis*

A. The expression of salivary protein genes in three stages of *S. chinensis*. FU: fundatrix, GL: fundatrigeniae, L2: nymphs.

| **Table S1**. The statistics of Hi-C data | |
| --- | --- |
| **Sample** | **Number** |
| **Raw Reads Number** | 738,531,166 |
| **Raw Bases Number** | 1.11E+11 |
| **Clean Reads Number** | 719,461,762 |
| **Clean Reads Rate (%)** | 97.42 |
| **Clean Bases Number** | 1.08E+11 |
| **Low-quality Reads Number** | 3,755,882 |
| **Low-quality Reads Rate(%)** | 0.51 |
| **Ns Reads Number** | 1,272,232 |
| **Ns Reads Rate(%)** | 0.17 |
| **Adapter Polluted Reads Number** | 14,041,290 |
| **Adapter Polluted Reads Rate(%)** | 1.9 |
| **Raw Q30 Bases Rate(%)** | 91.81 |
| **Clean Q30 Bases Rate(%)** | 92.06 |

**Table S2**. Statistics of the mapping rates on the genome assembly for genome sequencing data

| **Sample** | **Mean Depth** | **Coverage Rate (%)** | **Mapping Rate (%)** |
| --- | --- | --- | --- |
| *S. chinensis* | 60G | 99.70 | 97.79% |

| **Table S3**. Mapping rate of transcripts in the *Schlechtendalia chinensis* genome assembly | | | |
| --- | --- | --- | --- |
| **Sample** | **Insect stage** | **Genome mapping rate (%)** | **Gene set mapping rate (%)** |
| **AM** | Autumn migrant | 90.02% | 80.9 % |
| **FM** | Famale | 90.16% | 86.6% |
| **FU** | Fundatrix | 79.63% | 82.6% |
| **GL** | Fundatrigeniae | 90.23% | 81.4% |
| **L1** | Nymph | 88.29% | 82.8% |
| **L2** | Nymph | 88.98% | 80.2% |
| **MA** | Male | 79.38% | 84.5% |
| **MF** | Pregnant female | 85.01% | 88.9% |
| **SE** | Spring migrant | 85.65% | 89.6% |

| **Table S4**. *De novo* assembled transcript | |
| --- | --- |
| **Stat Type** | **Number** |
| **the genome contigs number** | 260,508 |
| **the longest length** | 26,864 |
| **the shortest length** | 190 |
| **the genome contig size** | 280,520,495 |
| **the rate of N** | 0 |
| **the rate of GC** | 0.382030019 |
| **the contig N50** | 2,028 |
| **the contig N90** | 402 |
| **the number of sequences >= 1kb** | 82,313 |
| **the number of sequences >= 2kb** | 40,897 |
| **the number of sequences >= 3kb** | 19,957 |

**Table S5** Statistical information of male and female reads mapping to chromosomes

| **Chromosome** | **Length** | **Female**  **MRPM** | **Male**  **MRPM** | **Female/Male Ratio** |
| --- | --- | --- | --- | --- |
| **chr01** | 79667931 | 7555508 | 8110550 | 0.93156543 |
| **chr02** | 20983503 | 2093863 | 2239691 | 0.934889232 |
| **chr03** | 20806501 | 2107514 | 2255576 | 0.934357344 |
| **chr04** | 20405002 | 2019769 | 2156499 | 0.936596307 |
| **chr05** | 19472003 | 1908887 | 2042550 | 0.934560721 |
| **chr06** | 19384925 | 1956508 | 2089950 | 0.936150626 |
| **chrX1** | 14859000 | 1439092 | 781901 | 1.840504105 |
| **chrX2** | 13144601 | 1333387 | 726210 | 1.836090112 |
| **chr07** | 11811579 | 1202491 | 1284708 | 0.936003356 |
| **chr08** | 11276004 | 1194436 | 1283928 | 0.930298272 |
| **chr09** | 11115576 | 1114298 | 1189328 | 0.936913955 |
| **chrX3** | 10827126 | 1051602 | 576946 | 1.822704378 |
| **chr10** | 10104278 | 1012412 | 1087178 | 0.931229293 |

| **Table S6**. Statistical results of repetitive sequences | | | |
| --- | --- | --- | --- |
| **Class** | **Element number** | **Length (bp)** | **% of genome** |
| **SINE** | 0 | 0 | 0% |
| **LINE** | 24,666 | 10,384,396 | 3.82% |
| **LTR elements** | 4,706 | 3,248,042 | 1.20% |
| **DNA elements** | 79,260 | 20,229,767 | 7.45% |
| **Unclassified** | 133,699 | 35,817,392 | 13.19% |
| **Small RNA** | 0 | 0 | 0 |
| **Satellites** | 32 | 2,214 | 0 |
| **Simple repeats** | 197,247 | 8,344,991 | 3.07% |
| **Low complexity** | 23,412 | 1,109,202 | 0.41% |
| **All** | 463,022 | 79,136,004 | 29% |

| **Table S7**. Genome annotation statistics of seven aphid genomes | | | | | | | |
| --- | --- | --- | --- | --- | --- | --- | --- |
| **Specie** | **Genome size (bp)** | **GC%** | **Total gene number** | **Average CDS length (bp)** | **Average exons per gene** | **Average exon length (bp)** | **Average intron length (bp)** |
| ***Acyrthosiphon pisum*** | 541,137,574 | 30% | 18,270 | 1,600 | 7.78 | 206 | 1,683 |
| ***Aphis glycines*** | 308,063,827 | 27% | 18,358 | 1,392 | 7.79 | 179 | 558 |
| ***Myzus persicae*** | 347,313,470 | 30% | 23,910 | 1,731 | 8.23 | 2,109 | 14,32 |
| ***Diuraphis noxia*** | 395,073,589 | 29% | 17,476 | 1,638 | 7.84 | 209 | 1,461 |
| ***Erisoma lanigerum*** | 334,868,377 | 26% | 28,186 | 990 | 4.13 | 240 | 756 |
| ***Rhopalosiphum maidis*** | 326,023,155 | 28% | 19,503 | 1,822 | 8.97 | 203 | 1,582 |
| ***Schlechtendalia chinensis*** | 280,452,825 | 34% | 14,089 | 1,535 | 7.26 | 212 | 910 |

| **Table S8**. Summary of ncRNA in *Schlechtendalia chinensis* | | |
| --- | --- | --- |
| **Type** | **Number** | **Total Length (bp)** |
| **tRNA** | 130 | 13,563 |
| **rRNA** | 29 | 16,764 |
| **miRNA** | 29 | 2,388 |
| **snRNA** | 72 | 9,093 |

**Table S9**. Go id description of GO enrichment analysis of expanded gene families in Eriosomatinaeand *Schlechtendalia chinensis*

| **Species** | **GO ID** | **Description** |
| --- | --- | --- |
| **Eriosomatinae** | **GO:0009812** | flavonoid metabolic process |
|  | **GO:0042178** | xenobiotic catabolic process |
|  | **GO:0042573** | retinoic acid metabolic process |
|  | **GO:0006787** | porphyrin-containing compound catabolic process |
|  | **GO:0006711** | estrogen catabolic process |
|  | **GO:0006789** | bilirubin conjugation |
|  | **GO:0009698** | phenylpropanoid metabolic process |
|  | **GO:0009804** | coumarin metabolic process |
|  | **GO:0046226** | coumarin catabolic process |
|  | **GO:0046271** | phenylpropanoid catabolic process |
|  | **GO:0051552** | flavone metabolic process |
|  | **GO:0052695** | cellular glucuronidation |
|  | **GO:0052696** | flavonoid glucuronidation |
|  | **GO:0052697** | xenobiotic glucuronidation |
|  | **GO:0070980** | biphenyl catabolic process |
|  | **GO:1904223** | regulation of glucuronosyltransferase activity |
|  | **GO:1904224** | negative regulation of glucuronosyltransferase activity |
|  | **GO:2001029** | regulation of cellular glucuronidation |
|  | **GO:2001030** | negative regulation of cellular glucuronidation |
|  | **GO:0018879** | biphenyl metabolic process |
| ***S. chinensis*** | **GO:0004666** | prostaglandin-endoperoxide synthase activity |
|  | **GO:0050473** | arachidonate 15-lipoxygenase activity |
|  | **GO:0000788** | nuclear nucleosome |
|  | **GO:0001550** | ovarian cumulus expansion |
|  | **GO:0008627** | intrinsic apoptotic signaling pathway in response to osmotic stress |
|  | **GO:0031620** | regulation of fever generation |
|  | **GO:0010335** | response to non-ionic osmotic stress |
|  | **GO:0031622** | positive regulation of fever generation |
|  | **GO:0032227** | negative regulation of synaptic transmission, dopaminergic |
|  | **GO:0048165** | fused antrum stage |
|  | **GO:0071471** | cellular response to non-ionic osmotic stress |
|  | **GO:0090360** | platelet-derived growth factor production |
|  | **GO:0090361** | regulation of platelet-derived growth factor production |
|  | **GO:0090362** | positive regulation of platelet-derived growth factor production |
|  | **GO:1902218** | regulation of intrinsic apoptotic signaling pathway in response to osmotic stress |
|  | **GO:1902219** | negative regulation of intrinsic apoptotic signaling pathway in response to osmotic stress |
|  | **GO:0001660** | fever generation |
|  | **GO:0010288** | response to lead ion |
|  | **GO:0031497** | chromatin assembly |
|  | **GO:0006333** | chromatin assembly or disassembly |

**TableS10**. Classification of salivary protein genes highly expressed in fundatrix

| **class** | **gene** | **Metabolic Pathway** | |
| --- | --- | --- | --- |
| **Detoxification** | Sc.chr01.1410 | Cytochrome P450 6CY18 | |
|  | Sc.chr03.0829 | Cytochrome P450 6k1 | |
|  | Sc.chr06.0408 | Heat shock protein 70 B2 | |
|  | Sc.chr07.301 | Cytochrome P450 6k1 | |
|  | Sc.chr10.021 | Glucose dehydrogenase | |
|  | Sc.chr01.0219 | Glucose dehydrogenase | |
|  | Sc.chr09.446 | UTP--glucose-1-phosphate uridylyltransferase | |
|  | Sc.chr06.0454 | Heat shock 70 | |
|  | Sc.chr06.0280 | Acetyl-CoA hydrolase; 4-hydroxybutyrate coenzyme A transferase | |
|  | Sc.chr06.0670 | Glutamate dehydrogenase | |
|  | Sc.chr03.0835 | 4-aminobutyrate aminotransferase | |
| **Signal transduction** | Sc.chr02.0173 | Ionotropic receptor 25a isoform X1 | |
|  | Sc.chr07.125 | Synaptotagmin 1 isoform X1 | |
|  | Sc.chr01.0800 | Guanine nucleotide-binding protein G(o) subunit alpha | |
|  | Sc.chr01.1250 | Adenylyl cyclase-associated protein 1 | |
|  | Sc.chr06.0154 | Inositol hexakisphosphate and Diphosphoinositol-pentakisphosphatekinase isoform X1 | |
|  | Sc.chr03.0344 | Synapse-associated protein of 47 kDa-like isoform X1 | |
| **Protein metabolism** | Sc.chr02.0275 | 26S proteasome regulatory subunit 4 | |
|  | Sc.chr02.0996 | Protein disulfide-isomerase | |
|  | Sc.chr03.1167 | Endoplasmin homolog | |
|  | Sc.chr04.0164 | Endoplasmic reticulum chaperone BiP isoform X1 | |
|  | Sc.chr01.0356 | Chitinase-like protein EN03 | |
| **Energy metabolism** | Sc.chr06.0491 | Rab GDP dissociation inhibitor alpha | |
|  | Sc.chr07.571 | Pyruvate kinase-like isoform X2 | |
|  | Sc.chr06.0789 | V-type proton ATPase subunit B | |
|  | Sc.chr01.1889 | ADP/ATP translocase 2 | |
| **Basic biological processes** | Sc.chr01.0974 | PiggyBac transposable element-derived protein 3 | |
|  | Sc.chr10.341 | Programmed cell death protein 4 | |
|  | Sc.chr03.1161 | Dihydrolipoyl dehydrogenase, mitochondrial | |
|  | Sc.chr10.506 | TOM1-like protein 2 | |
|  |  | | |
| **Movement** | Sc.chr03.1184 | Tubulin alpha-1C chain | |
|  | Sc.chrX1.195 | Tubulin beta chain | |
|  | Sc.chr09.365 | Troponin T isoform X8 | |
|  | Sc.chr04.0096 | Tubulin beta chain-like |  |
| **Function unknow** | Sc.chrX2.133 |  |  |
